# Pseudogenization of a Sweet-Receptor Gene Accounts for Cats' Indifference toward Sugar

**DOI:** 10.1371/journal.pgen.0010003

**Published:** 2005-07-25

**Authors:** Xia Li, Weihua Li, Hong Wang, Jie Cao, Kenji Maehashi, Liquan Huang, Alexander A Bachmanov, Danielle R Reed, Véronique Legrand-Defretin, Gary K Beauchamp, Joseph G Brand

**Affiliations:** 1 Monell Chemical Senses Center, Philadelphia, Pennsylvania, United States of America; 2 The WALTHAM Centre for Pet Nutrition, Melton Mowbray, Leicestershire, United Kingdom; 3 Department of Psychology, School of Arts and Sciences and Department of Anatomy, School of Veterinary Medicine, University of Pennsylvania, Philadelphia, Pennsylvania, United States of America; 4 Department of Biochemistry, School of Dental Medicine, University of Pennsylvania, Philadelphia, Pennsylvania, United States of America; 5 Veterans Affairs Medical Center, Philadelphia, Pennsylvania, United States of America; University of Oxford, United Kingdom

## Abstract

Although domestic cats *(Felis silvestris catus)* possess an otherwise functional sense of taste, they, unlike most mammals, do not prefer and may be unable to detect the sweetness of sugars. One possible explanation for this behavior is that cats lack the sensory system to taste sugars and therefore are indifferent to them. Drawing on work in mice, demonstrating that alleles of sweet-receptor genes predict low sugar intake, we examined the possibility that genes involved in the initial transduction of sweet perception might account for the indifference to sweet-tasting foods by cats. We characterized the sweet-receptor genes of domestic cats as well as those of other members of the Felidae family of obligate carnivores, tiger and cheetah. Because the mammalian sweet-taste receptor is formed by the dimerization of two proteins (T1R2 and T1R3; gene symbols *Tas1r2* and *Tas1r3*), we identified and sequenced both genes in the cat by screening a feline genomic BAC library and by performing PCR with degenerate primers on cat genomic DNA. Gene expression was assessed by RT-PCR of taste tissue, in situ hybridization, and immunohistochemistry. The cat *Tas1r3* gene shows high sequence similarity with functional *Tas1r3* genes of other species. Message from *Tas1r3* was detected by RT-PCR of taste tissue. In situ hybridization and immunohistochemical studies demonstrate that *Tas1r3* is expressed, as expected, in taste buds. However, the cat *Tas1r2* gene shows a 247-base pair microdeletion in exon 3 and stop codons in exons 4 and 6. There was no evidence of detectable mRNA from cat *Tas1r2* by RT-PCR or in situ hybridization, and no evidence of protein expression by immunohistochemistry. *Tas1r2* in tiger and cheetah and in six healthy adult domestic cats all show the similar deletion and stop codons. We conclude that cat *Tas1r3* is an apparently functional and expressed receptor but that cat *Tas1r2* is an unexpressed pseudogene. A functional sweet-taste receptor heteromer cannot form, and thus the cat lacks the receptor likely necessary for detection of sweet stimuli. This molecular change was very likely an important event in the evolution of the cat's carnivorous behavior.

## Synopsis

Although sweet sugars are ubiquitous in human foods, they are seldom added to cat food, and owners usually do not feed sweets to their cats. This is because, in contrast to most other mammals, both domestic cats and their wild cousins, the big cats, do not show a preference for and, most likely, cannot detect sweet-tasting compounds. Other than this sweet blindness, the cat's sense of taste is normal. The molecular mechanism for this unique behavior towards sweets was not known, until now. Sweet compounds, including sugars and artificial sweeteners, are recognized by a special taste bud receptor composed of the products of two genes. The authors found that in cats, one of these genes is not functional and is not expressed. (It is called a pseudogene.) Because the sweet receptor cannot be formed, the cat cannot taste sweet stimuli. During the evolution of the cats' strictly carnivorous behavior, selection to maintain a functional receptor was apparently relaxed. This research provides a molecular explanation for the common observation that the cat lives in a different sensory world than the cat owner.

## Introduction

The domestic cat (*Felis silvestris catus*), of the family Felidae in the order Carnivora, is an obligate carnivore. Its sense of taste is distinguished by a lack of attraction to, or indifference toward, compounds that taste sweet to humans, such as sweet carbohydrates (sugars) and high-intensity sweeteners [1–3]. This behavior toward sweet stimuli is in marked contrast to the avidity for sweets shown by most omnivores and herbivores and even some other carnivores such as the dog [4]. The indifference that cats display toward sweet-tasting compounds contrasts with their otherwise normal taste behavior toward stimuli of other taste modalities. For example, they show preference for selected amino acids [5] and generally avoid stimuli that to humans taste either bitter or very sour [1,5]. Congruent with these behavioral responses to taste stimuli, recordings from cat taste nerve fibers, and from units of the geniculate ganglion innervating taste cells, demonstrate responses to salty, sour, and bitter stimuli as well as to amino acids and nucleotides, but do not show neural responses to sucrose and several other sugars [5–12]. The sense of taste in the cat, in general, is therefore similar to that of other mammals, with the exception of an inability to taste sweet stimuli.

The molecular basis for this sweet blindness in cats is not known. Because the taste blindness appears specific to this single modality, we postulated that the defect in the cat (and likely in other obligate carnivores of Felidae) lies at the receptor step subtending the sweet-taste modality. The possible defects at the molecular level that might cause this sweet blindness could range from a single to a few amino acid substitutions, such as is found between sweet “taster” and “nontaster” strains of mice [13,14], to more radical mechanisms, such as an unexpressed pseudogene.

To distinguish among these possibilities, we identified the DNA sequence and examined the structures of the two known genes, *Tas1r2* and *Tas1r3,* that in other mammals encode the sweet-taste receptor heteromer, T1R2/T1R3. We compared these with the sequence and structure of the same genes in dog, human, mouse, and rat—all species that display a functional sweet-taste modality. We also sought to detect the expression of the two cat genes at both the RNA and protein levels. Our results lead us to conclude that *Tas1r3* is expressed in cat taste buds and very likely is functional, whereas cat *Tas1r2* is an unexpressed pseudogene. The immediate repercussion of this unexpressed gene is that the heteromer normally acting as a sweet-taste receptor in most other mammals likely does not form in the cat.

## Results

We identified DNA sequences of *Tas1r3* and *Tas1r2* from the domestic cat by screening a feline BAC library and using a PCR strategy on cat genomic DNA with degenerate primers. The feline sequences were compared with those of other species, and gene structures were determined. The expression of these two receptors was then evaluated by in situ hybridization and immunohistochemistry.

### Molecular Cloning of Cat *Tas1r3* and *Tas1r2*: Sequence and Gene Structure

**Figure 1:**
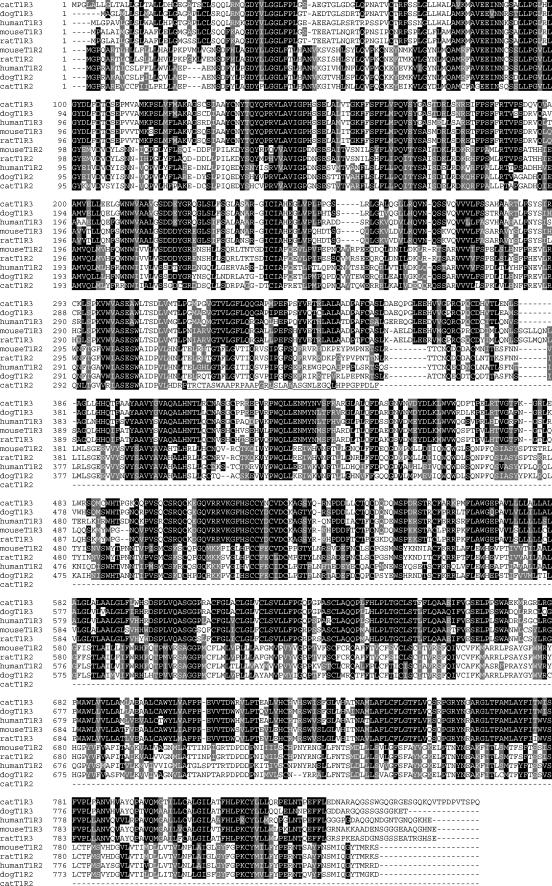
Alignment of Deduced Amino Acid Sequences of T1R3 and T1R2 from Five Species This figure shows the alignment of the deduced sequences of the taste receptor proteins, T1R3 and T1R2, from domestic cat, domestic dog, human, mouse, and rat. Amino acids that are identical among species are shaded in black; conservative amino acid substitutions are shaded in gray. The cat T1R3 sequence shows high similarity with that of human and rodents, with especially high similarity with that of dog. The predicted cat T1R2 sequence is truncated at amino acid 355 due to a premature stop codon at bp 57–59 in exon 4, which results from a 247-bp deletion in exon 3. The underlined amino acids from 316 to 355 of the cat T1R2 result from the frame shift brought by the 247-bp deletion in exon 3. Note that the deduced amino acid sequence of dog T1R2 predicts an apparently normal protein showing high similarity with that of rat, mouse, and human.

By sequencing positive BAC clones retrieved from a feline genomic BAC library (*Felis silvestris catus*; BACPAC Resources, Oakland, California, United States), we obtained more than 3 kb of genomic sequences containing the open reading frame for cat *Tas1r3*, and approximately 10 kb of genomic sequences containing the open reading frame for cat *Tas1r2*. Because exons 1 and 2 of *Tas1r2* were not found in the positive BAC clones, we employed a PCR strategy using degenerate primers to amplify these regions from cat genomic DNA (Novagen, San Diego, California, United States) (See Materials and Methods). We aligned the cDNA sequences and the deduced amino acid sequences from cat *Tas1r3* and *Tas1r2* with their dog, human, mouse, and rat orthologs (Figure 1). (We obtained the sequences of domestic dog genes, *Tas1r3* and *Tas1r2,* by screening a dog genomic library using the same overgo probes and methods as for the feline genomic BAC library and by taking advantage of the limited data available at that time from the public dog genome database at http://www.ncbi.nlm.nih.gov/genome/guide/dog/).

**Figure 2:**
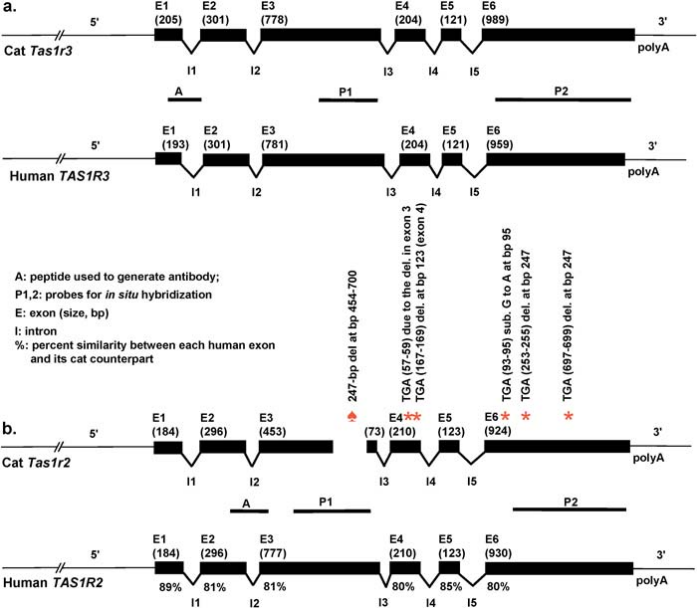
Gene Structures of Cat *Tas1r3,* Human *TAS1R3*, and Cat *Tas1r2*, Human *TAS1R2* The exons are shown in black (size in bp of each exon is in parentheses). Boundaries of gene sequences used to produce probes for in situ hybridization studies (Figure 3) are shown by the horizontal lines labeled “P1” and “P2” under the sketch of the cat *Tas1r3* and cat *Tas1r2*. Boundaries of sequence used to generate peptide antigens for immunohistochemical studies (Figure 4) are shown by the horizontal lines labeled “A” under the sketch of the cat *Tas1r3* and cat *Tas1r2.* The locations referred to in the vertical explanation text above the asterisks and the spade symbol indicate the position in bp within each exon. Intron sizes shown in the figure are not proportionally scaled on both (A) and (B) because of the large size of *Tas1r2* introns. Under each human exon is the percent similarity between each human exon and its cat counterpart at the nucleotide level (Figure 2B). The exons for cat *Tas1r2* refer to parts corresponding to human exons. The spade symbol (♠) indicates the position of microdeletion in exon 3 of cat *Tas1r2*. Asterisks (*) indicate the stop codon positions in exon 4 and 6 of cat *Tas1r2*. Note that nucleotide numbers of the exon 3 in human *TAS1R2* and cat *Tas1r2* are not identical.

Table 1 presents the percent similarity of the *Tas1r3* and *Tas1r2* genes at both the cDNA and the protein levels between all possible pairs of five species: cat, dog, human, mouse, and rat. The cat *Tas1r3* gene shows high similarity at the cDNA level with that of dog (87%), human (79%), rat (75%), and mouse (74%) (Table 1). The cat *Tas1r3* gene predicts a protein of 865 amino acids (Figure 1) showing 85% similarity with deduced protein of dog, and 73%, 72%, and 72% with that of human, mouse, and rat, respectively (Table 1). Initially we predicted the exon–intron boundaries of cat *Tas1r3* by comparison with the known boundaries of human *TAS1R3*. To confirm these exon–intron boundaries for cat *Tas1r3*, we performed both RT-PCR on cDNA from cat taste bud–containing circumvallate and fungiform papillae, and PCR on cat genomic DNA using intron-spanning primers. By comparing the cDNA sequence with the genomic sequence, we confirmed the boundaries predicted from human *TAS1R3* (Figure 2A). Both the cat *Tas1r3* and human *TAS1R3* genes are composed of six similarly sized exons and five introns (Figure 2A).

**Table 1:**
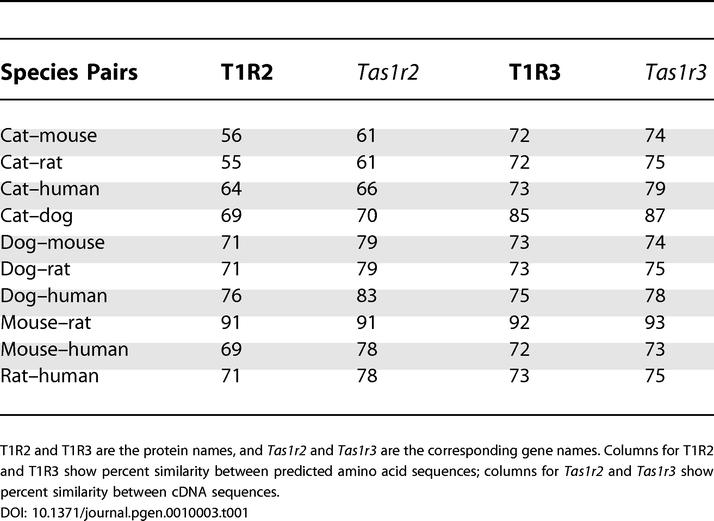
Similarity of Sweet Receptors between Species

T1R2 and T1R3 are the protein names, and *Tas1r2* and *Tas1r3* are the corresponding gene names. Columns for T1R2 and T1R3 show percent similarity between predicted amino acid sequences; columns for *Tas1r2* and *Tas1r3* show percent similarity between cDNA sequences.

**Figure 3:**
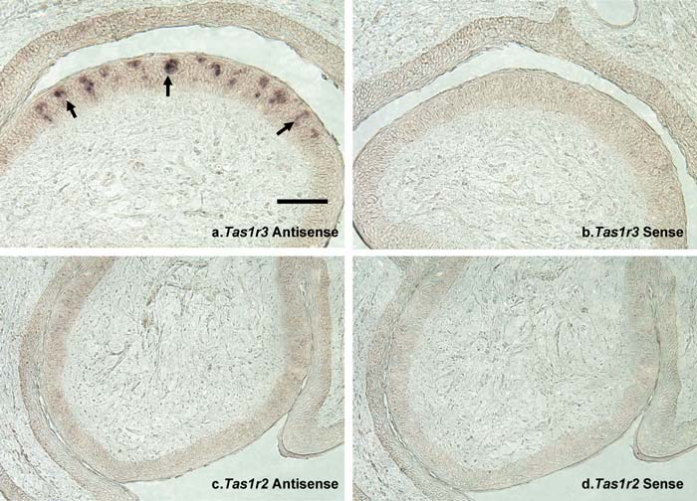
RNA Expression of Cat *Tas1r2* and *Tas1r3* from Circumvallate Papillae Digoxigenin-labeled sense and antisense cRNA probes corresponding to exons 3 and 6 of cat *Tas1r2* and *Tas1r3* were synthesized using DIG RNA labeling kit (Roche Applied Science, Indianapolis, Indiana, United States) (See Figure 2 for the locations of in situ probes, and Table 3 for identity of primers.) Hybridizations were carried as described [39]. Panel (A) shows result of antisense probes for *Tas1r3*, whereas panel (B) shows the result of the sense probes for *Tas1r3*. Panel (C) shows results of the antisense probes for *Tas1r2* whereas panel (D) shows results of the sense probes. Scale bar, shown only in panel (A), = 60 μm for (A), (B), (C), and (D).

**Figure 4:**
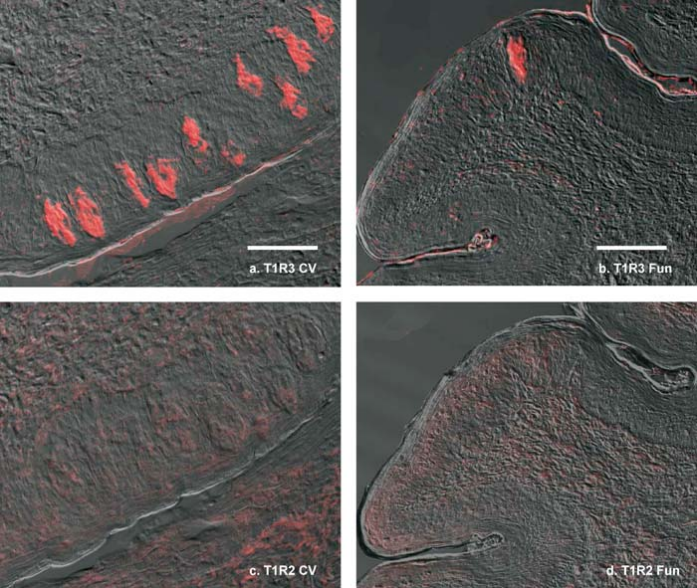
Protein Expression of Cat T1R2 and T1R3 Cat T1R3 expression is detected in taste buds of circumvallate papilla (CV) (A) and a fungiform papilla (Fun) just anterior to the intermolar eminence (B) by labeling with anti-mouse T1R3 antibody. Cat T1R2 expression is not detectable in either circumvallate (C) or fungiform (D) using an anti-cat T1R2 antibody. Control studies demonstrated that the anti-cat T1R2 antibody labeled a subset of taste bud cells in rat circumvallate (data not shown). Scale bar, shown only in panel (A) and (B), = 60 μm for (A) and = 45 μm for (B). Scale for panel (C) is the same as that of panel (A); scale for panel (D) is the same as that of panel (B).

We identified the exon–intron boundaries of cat *Tas1r2* by comparison with known human boundaries (Figure 2B). Examining the sequence of cat *Tas1r2,* we discovered a microdeletion of 247 base pairs (bp) within exon 3. This deletion is responsible for a frame shift that results in a premature stop codon at bp 57–59 of exon 4 (Figure 2B). Assuming, for the moment, that a protein is translated from cat *Tas1r2,* then, because of the deletion and premature stop codon, the gene sequence predicts a peptide of 355 amino acids, the first 315 of which show high similarity with their rat, mouse, human, and dog counterparts (see Figure 1). Because of the frame shift introduced by the 247-bp deletion, the remaining deduced 40 amino acids show no similarity with their rat, mouse, human, or dog counterparts (underlined sequence of cat T1R2; Figure 1). The predicted similarity of this hypothetical 355–amino acid protein was compared with its truncated counterparts from dog, human, mouse, and rat. It ranges from 55% to 69% (Table 1). In contrast, the percent similarity of the full-length T1R2 protein within pairs of other species is between 91% (mouse–rat) and 69% (mouse–human).

By aligning cat *Tas1r2* DNA sequences of exons 4, 5, and 6 with their human counterparts, we found four additional stop codons: one in exon 4 due to a deletion at bp 123, and three in exon 6 due to a substitution at bp 95 and a deletion at bp 247 (Figure 2B). The multiple stop codons indicate that the cat *Tas1r2* is a pseudogene.

In an attempt to confirm the cat *Tas1r2* exon–intron boundaries, we performed RT-PCR on cDNA from cat circumvallate and fungiform taste papillae. Despite using numerous (> 70) primers corresponding to deduced message from the *Tas1r2* gene, we were unable to detect it.

### RNA and Protein Expression

Having detected message from cat *Tas1r3,* but not from cat *Tas1r2,* by RT-PCR, we used the more tissue-specific approaches of in situ hybridization and immunohistochemistry to refine the search for cat *Tas1r2* gene expression, using the cat *Tas1r3* gene for comparison. Probes for in situ hybridization were constructed from the gene sequences corresponding to the lines marked “P” in Figures 2A and 2B. (See Materials and Methods for details.) Figure 3 shows that message from *Tas1r3* is expressed in taste buds of cat circumvallate papillae whereas *Tas1r2* expression is not detectable by in situ hybridization. Antisense probes for *Tas1r3* result in positive labeling (Figure 3A); the arrows indicate three of the many labeled taste buds. The control sense probes show no labeling (Figure 3B). In contrast, antisense probes for cat *Tas1r2* show no detectable labeling (Figure 3C) as is the case for the sense control (Figure 3D).

To detect the presence of taste receptor proteins from *Tas1r2* and from *Tas1r3,* we exposed 10-μm sections of cat circumvallate and fungiform papillae to polyclonal antibodies developed against deduced amino acid peptide antigens marked by the line labeled “A” in Figure 2A and 2B. T1R3-like immunoreactivity was present in the taste buds of every circumvallate (10) and fungiform (4) papilla used in this study (Figure 4A and 4B) whereas immunoreactivity to T1R2 was not detected in these same tissues (Figure 4C and 4D). (Each circumvallate papilla of the cat contains approximately 400 taste buds, whereas the large fungiform papillae used in this study, located in the area of the eminence, contain from 1 to about 15 taste buds each.) The antibody to cat T1R2 did, however, label a subset of taste buds in rat circumvallate papillae (results not shown).

### Confirmation of *Tas1r2* Sequence in Six Individual Cats, Tiger, and Cheetah

Because the feline BAC genomic library was constructed from a single individual cat, we confirmed the sequence of *Tas1r2* in six additional, unrelated, healthy adult domestic cats. Genomic DNA was obtained by cheek swabs from five of the six cats and through a blood sample from the remaining cat, amplified by PCR using primers that flanked the deletion and stop codons of the known cat *Tas1r2,* and sequenced. In addition, to test whether other species of Felidae display similar sequence anomalies in their *Tas1r2* gene, we performed PCR on genomic DNA of one tiger (Therion International, Saratoga Springs, New York, United States) and one cheetah (a gift from the San Diego Zoo). We found that *Tas1r2* in all six cats, the tiger, and the cheetah show the identical 247-bp deletion in exon 3, and all have stop codons at the same positions in exon 4 (Table 2). In exon 6, we found evidence for two alleles at position 93–95 in domestic cat, wherein two cats show the stop codon, TGA (homozygotes TGA/TGA); one cat shows TGR (heterozygote TGA/TGG); and three of the domestic cats, the one tiger, and the single cheetah show TGG (homozygotes TGG/TGG) (Table 2). The second exon 6 stop codon is also common to all three species (TGA for domestic cat, TAG for tiger and cheetah). Although the third stop codon of exon 6 at bp 697–699 was found in all six domestic cats, the corresponding region in tiger and cheetah could not be amplified by PCR.

**Table 2:**
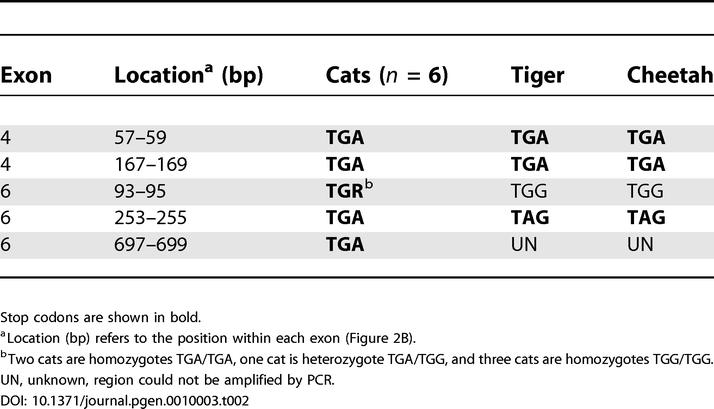
*Tas1r2* Stop Codons in Species of Felidae

Stop codons are shown in bold.

^a^Location (bp) refers to the position within each exon (Figure 2B).

^b^Two cats are homozygotes TGA/TGA, one cat is heterozygote TGA/TGG, and three cats are homozygotes TGG/TGG.

UN, unknown, region could not be amplified by PCR.

Collectively, these data indicate that cat *Tas1r3* is an expressed and likely functional receptor, whereas cat *Tas1r2* is an unexpressed pseudogene.

## Discussion

The taste receptors for sweetness and for umami (an amino acid–taste modality) are members of the T1R family of taste receptors [15,16,17]. These are Class C, family 3, G protein–coupled receptors (GPCR). The three known members of the T1R family are T1R1, T1R2, and T1R3 (for review, see [18]). In rodents and primates the primary sweet-taste receptor is composed of a dimer of two closely related GPCRs, T1R2 and T1R3 [14,15,16,17].

For this study, we made the working assumption that the Felidae T1R family shows specificity similar to that known from rodents and primates. Because the umami receptor is composed of the heteromer, T1R1/T1R3, and because cats can taste amino acids, it would appear likely that both of these proteins should be functional. The sweet-taste receptor is composed of the heteromer T1R2/T1R3. Because the cat cannot taste sweet stimuli, the most likely assumption is that the cat T1R2 is non-functional.

### Molecular Features of Cat *Tas1r3*

By comparison with other known T1R3 proteins and other proteins of Class C, family 3, the sequence and gene structure of cat *Tas1r3* predict a functional receptor of 865 amino acids (see Figure 1). Cat *Tas1r3* is assumed to be located on cat Chromosome C1, syntenic with human 1p36, where human *TAS1R3* is located [19,20]. As with other *Tas1r3* genes, the cat *Tas1r3* is composed of six exons, each approximately the same size as those of human (see Figure 2A). The sequence of cat *Tas1r3* predicts a seven-transmembrane GPCR with extended N-terminal domain (first transmembrane region spanning amino acids 572–595), features common to other T1R3 receptors. Important Class C, family 3, structural motifs can also be located in cat T1R3 including the xPKxY motif at amino acids 814–818, and the FHSCCY motif at amino acids 517–522. Additionally, although most members of Class C, family 3, GPCRs show a highly conserved arginine residue at the extreme 3′ end of transmembrane segment 3, an exception is found with human, mouse, and rat T1R3, which substitute glutamic acid (E) for arginine (R) [21]. This substitution is also found in cat T1R3 at amino acid 660 (see Figure 1; the deduced dog T1R3 substitutes glutamine (Q) for arginine at the end of TM3).

Available evidence indicates that the products of cat *Tas1r3* are expressed in taste buds. RT-PCR readily detected the message from *Tas1r3* in lingual taste bud–containing tissues (results not shown). In situ hybridization studies confirmed the presence of message and localized it to taste buds (see Figure 3A). Polyclonal antibodies developed against T1R3 labeled taste buds in both cat circumvallate (Figure 4A) and fungiform papillae (Figure 4B). While only a few cells showed evidence of T1R3-like immunoreactivity, nearly every taste bud was labeled by both in situ hybridization and immunohistochemistry.

These commonalities in gene structure and sequence, together with evidence that the cat *Tas1r3* gene is expressed, are consistent with the assumption that cat *Tas1r3* codes for a functional receptor.

### Molecular Features of Cat *Tas1r2*

Cat *Tas1r2,* on the other hand, while retaining structure similar with that of the human *TAS1R2* gene (see Figure 2B), is an unexpressed pseudogene. The likely important molecular event that resulted in cat *Tas1r2* becoming a pseudogene is the 247-bp deletion in exon 3. This deletion results in a frame shift that brings about a premature stop codon in exon 4 (Figure 2B). An additional stop codon can be found in exon 4, with three more in exon 6 (Figure 2B). This apparent accumulation of mutations suggests that there is no pressure from natural selection on the cat *Tas1r2* gene. To determine if this gene is expressed, we performed studies to detect message from cat *Tas1r2* by RT-PCR of taste bud–containing lingual papillae and by in situ hybridization. For RT-PCR, numerous (>70) primers were constructed based on sequences from exons 1–6. For in situ hybridization, probes were designed from exon 3 and from exon 6 (Figure 2B**;** Table 3). Both techniques failed to detect message from cat *Tas1r2* (see Figure 3C and 3D). Consistent with these attempts to detect message from cat *Tas1r2,* immunohistochemistry using an antibody developed from a deduced amino acid sequence spanning exons 2 and 3 revealed no labeling of taste buds in circumvallate or fungiform papillae (Figure 4C and 4D).

These results suggest that the cat *Tas1r2* pseudogene is not transcribed, or if it is transcribed, it rapidly degrades, perhaps through a nonsense-mediated mRNA decay pathway [22].

**Table 3:**
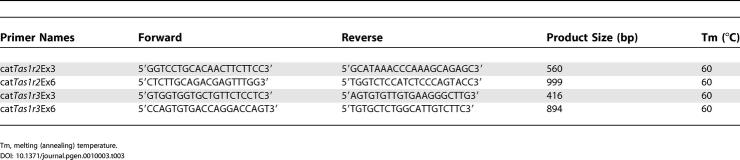
Primers for In Situ Probes

Tm, melting (annealing) temperature.

### *Tas1r2* in Felidae

The generality of the pseudogene nature of cat *Tas1r2* was confirmed by sequencing the deletion and stop codon areas from six individual healthy adult cats. All showed the deletion and similar stop codons with some polymorphism (see Table 2). To assess the generality of the pseudogene nature of *Tas1r2* in Felidae, we sequenced the stop codon areas and the area including the exon 3 microdeletion from genomic DNA of tiger and cheetah. These too displayed microdeletion and stop codons at the same location as the domestic cat. These observations, suggesting that in at least three species of Felidae *Tas1r2* is not expressed, are consistent with behavioral evidence showing that, not only domestic cats, but also tigers and cheetahs do not prefer sweetened water over plain water [1].

According to morphological and molecular evidence, the available phylogeny of the order Carnivora consists of two groups, the Feliformia (cats, mongooses, civets, and hyenas) and the Caniformia (wolves, bears, raccoons, mustelids, and pinnipeds) [23,24]. It is difficult to determine when the alteration of *Tas1r2* occurred and whether it preceded or followed the cat ancestor's change in diet to exclude plants. Clearly, because dogs have a human-like T1R2 structure (see Figure 1) and an avidity for sweet carbohydrates [25], the changes in the cat *Tas1r2* must have occurred after the divergence of the Feliformia and the Caniformia.

### Genes Affecting Taste Behavior

Taste receptors are shaped by and reflect a species' food choices. The genes encoding taste receptors often show a good deal of variation both among species and among individuals. These variations, both subtle and obvious, can have a variety of effects on taste sensitivity and preference behavior. A textbook example of this effect is the individual variation seen in sensitivity to the bitter compound, phenylthiocarbamide (PTC). A gene of the human *TAS2R* family of bitter taste receptors, *TAS2R38,* associated with this individual variation, shows three coding single-nucleotide polymorphisms giving rise to five haplotypes worldwide, accounting for the 55% to 85% of the variance in PTC sensitivity [26]. Further, in Drosophila, the behavioral and electrophysiological responses to trehalose are diminished in two mutants that carry deletions in the trehalose recognition gene, *Gr5a* [27]. In the mouse, variation in preference for sweet-tasting stimuli maps to the gene for T1R3, located within the *Sac* locus [28,29]. This gene is allelic in mice, and several reports identify a missense mutation (I60T) as being the most likely mutation accounting for the phenotypic differences [13,14,16,30–33]. However, the same alleles are not involved in strain-dependent sweet-taste preference in rats [34].

In addition to the modulation of behavior that can be caused by point mutations, more profound behavioral changes can result from the abolishment of gene function through, for example, the generation of pseudogenes. An example of this effect in mammalian chemoreception lies within the large repertoire of olfactory receptor genes. More than 60% of the human olfactory receptor genes are pseudogenes [35], whereas, only 20% are classified as such in mouse [35,36]. Strikingly, the accumulation of these olfactory pseudogenes in primates reportedly occurred concomitant with the acquisition of trichromatic color vision, perhaps reflecting the overarching behavioral changes that such an acquisition engendered [37]. Similar generation of bitter-taste receptor pseudogenes, accompanied by a large number of coding region single-nucleotide polymorphism, can account for the broad diversity displayed by the bitter-taste receptor family. This diversity may possibly play an important role in both species-specific and individually manifested taste preference [38].

In the extreme case, where a species fails to respond to stimuli representative of an entire modality, such as the cat with sweet taste, the development of a unique food preference behavior, based on the remaining taste receptors, might be anticipated. Because, with the exception of the sweetness modality, the taste system of the cat is organized much like that of most other mammals, discovering the molecular basis for the cats' lack of response to sweet-tasting compounds gives us a window on the development of strict carnivorous behavior in Felidae.

### Conclusion

It is known that Felidae do not detect sweetness of carbohydrates yet can taste amino acids. Our results indicate that the gene encoding one member of the sweet-taste receptor heteromer is an unexpressed pseudogene. Given this observation, we suggest that the most parsimonious explanation for the inability of Felidae to respond to sweeteners is the lack of a functional T1R2 protein.

## Materials and Methods

### 

#### Animal tissue.

We obtained cat taste tissue from healthy young-adult animals euthanized for reasons unrelated to this study. Animals were cared for under protocols 033400 and 057600 approved by the Institutional Animal Care and Use Committee of the University of Pennsylvania to Dr. Mark Haskins of the School of Veterinary Medicine, University of Pennsylvania.

#### Preparation of overgo probes.

Overgo probes are comprised of two 22mers with a complementary eight-base overlap. They can be designed by a computer program (http://genome.wustl.edu/tools/?overgo=1) and are readily synthesized. To identify cat *Tas1r2* and *Tas1r3,* overgo probes were designed by aligning conserved coding regions of *Tas1r2* and *Tas1r3* sequences from different species. The single-stranded overhangs (14 bases) were filled in with ^33^P-labeled dATP and dCTP, and the overgo probes were used in hybridization procedures with the BAC libraries.

#### Screening a feline genomic BAC library.

*Tas1r2* and *Tas1r3* overgo probes were radioactively labeled by the random hexa-nucleotide method, and hybridization and washing of membranes were as described [29]. We identified 47 positive BAC clones for cat *Tas1r2* and cat *Tas1r3,* and sequenced all of the positive BAC ends. By aligning BAC ends sequences with human syntenic regions (human *TAS1R2* and *TAS1R3* are located on chromosome 1p36), we picked BAC clones positive for cat *Tas1r2* and *Tas1r3* for shotgun library preparation.

#### Production of shotgun libraries for BACs containing cat *Tas1r2* and *Tas1r3*.

We prepared BAC DNAs from positive clones by using a Qiagen Large Construct Kit (Valencia, California, United States). The BAC DNAs were digested using Sau3A I and the digested BAC DNA fragments subcloned into pGEM-3Z (Promega) vector. After transformants were arrayed to a nylon membrane, two separate hybridizations were performed by using pooled *Tas1r2* and *Tas1r3* overgo probes. By sequencing positive clones from the shotgun libraries and by using a chromosome walking strategy, we obtained the full coding region of the cat *Tas1r3* and exon 3 to exon 6 of cat *Tas1r2*.

#### Identification of exon 1 and exon 2 of the cat *Tas1r2* by PCR strategy.

Because exon 1 and exon 2 of the cat *Tas1r2* were not present in the positive BACs selected above, we designed degenerate primers based on *Tas1r2* alignments from different species (human, rodents, and dog) and performed PCR using cat genomic DNA as a template. The PCR products were sequenced. The feline BAC library was then re-screened using PCR products as probes, and new positive BAC clones were retrieved. Using a chromosome walking strategy, we obtained the complete sequence of exon 1 and exon 2 of cat *Tas1r2* from these newly retrieved BAC clones.

#### RT-PCR.

To examine the RNA expression and to determine the intron–exon boundaries of the cat *Tas1r2* and *Tas1r3* genes, we extracted total RNA using TRIZOl Reagent (Life Technologies Inc., Rockville, Maryland, United States) from cat taste bud–containing tissues, followed by reverse transcription (Superscript reverse transcriptase, Life Technologies). The cDNA samples were amplified using AmpliTaq DNA Polymerase with GeneAmp (Perkin Elmer Corporation, Branchburg, New Jersey, United States) and intron-spanning primers selected to distinguish genomic and cDNA. Single bands of expected sizes were excised from the gel, purified, and sequenced.

#### In situ hybridization.

The probes corresponding to exons 3 and 6 of cat *Tas1r2* and *Tas1r3* were amplified by PCR using the primers described in Table 3. Digoxigenin-labeled cRNA probes were synthesized using a DIG RNA labeling kit (Roche). Taste bud–containing vallate tongue tissue was obtained as above. Fresh frozen sections (14 μm/section) of cat circumvallate papillae were attached to clean SuperFrost/Plus slides and prepared for in situ hybridization [39]. High-stringency hybridizations were carried out at 70 °C overnight in 50% formamide, 5X SSC, 5X Denhardt's, 250 μg/ml yeast RNA, and 500 μg/ml sperm DNA using the mixed cRNA probes. Sections were washed at 72 °C with 0.2X SSC three times. Signals were detected using alkaline phosphatase–conjugated antibodies to digoxigenin and standard chromogenic substrates and observed with a Nikon SA Microphot Microscope. Control hybridizations were performed with sense probes.

#### Immunohistochemistry.

Polyclonal anti-cat T1R2 rabbit antisera directed against an N-terminal peptide of cat T1R2 (exons 2 and 3; see Figure 1) were generated by Zymed Laboratories, Inc. (South San Francisco, California, United States). Generation of antisera directed against N-terminal peptide of mouse T1R3 has been described previously [33]. Lingual tissue blocks containing cat circumvallate and fungiform papillae were fixed in 4% paraformaldehyde for 2–6 h, then processed [40]. The antibodies were incubated with the sections (10 μm/section) for 60 h at 4 °C. After washing, the sections were incubated with the secondary antibody (Cy3-conjugated goat anti-rabbit IgG; The Jackson Laboratory, Bar Harbor, Maine, United States) and observed with a Leica TCS SP2 Spectral Confocal Microscope (Leica Microsystems Inc., Mannheim, Germany). Single-channel fluorescence images (average projection of 20–25, 0.3-μm optical sections) were processed with Adobe Photoshop software and overlaid on their respective difference interference contrast images.

#### Examination of stop codons in six individual cats and other species within Feliformia.

To confirm that *Tas1r2* is a pseudogene in other cats, we obtained genomic DNA from cheek swabs or blood of six unrelated healthy adult cats. We sequenced the areas around the microdeletion and the stop codons by PCR using primers that flanked these areas of interest. In addition, to test whether other species of Felidae have a functional *Tas1r2* gene, we performed PCR on genomic DNA of one tiger (Therion International, Saratoga Springs, New York, United States) and one cheetah (a gift from the San Diego Zoo) using the same primers above. All the PCR products are purified and sequenced.

## Supporting Information

### Accession Numbers

The GenBank (http://www.ncbi.nlm.nih.gov/Genbank/) accession numbers for the genomes discussed in this paper are cat *Tas1r3* (AY819786), cat *Tas1r2* (AY819787), dog *Tas1r2* (AY916758), dog *Tas1r3* (AY916759), human *TAS1R3* (BK000152), and human *TAS1R2* (NM_152232)*.*

## Abbreviations

bp: base pair
GPCR: G protein–coupled receptors

## References

[pgen-0010003-b01] Beauchamp GK, Maller O, Rogers JG (1977). Flavor preferences in cats (Felis catus and Panthera sp.). J Comp Physiol Psychol.

[pgen-0010003-b02] Carpenter JA (1956). Species differences in taste preferences. J Comp Physiol Psychol.

[pgen-0010003-b03] Bartoshuk LM, Jacobs HL, Nichols TL, Hoff LA, Ryckman JJ (1975). Taste rejection of nonnutritive sweeteners in cats. J Comp Physiol Psychol.

[pgen-0010003-b04] Bradshaw JW (1991). Sensory and experiential factors in the design of foods for domestic dogs and cats. Proc Nutr Soc.

[pgen-0010003-b05] White TD, Boudreau JC (1975). Taste preferences of the cat for neurophysiologically active compounds. Physiol Psychol.

[pgen-0010003-b06] Boudreau JC, Bradley BE, Bierer PR, Kruger S, Tsuchitani C (1971). Single unit recordings from the geniculate ganglion of the facial nerve of the cat. Exp Brain Res.

[pgen-0010003-b07] Boudreau J, Alev N (1973). Classification of chemoresponsive tongue units of the cat geniculated ganglion. Brain Res.

[pgen-0010003-b08] Boudreau JC (1977). Chemical stimulus determinants of cat neural taste responses to meats. J Am Oil Chem Soc.

[pgen-0010003-b09] Dinger B, Fidone SJ, Stensaas FJ (1984). Gustatory trophic action of arterial chemosensory neurones in the cat. J Physiol.

[pgen-0010003-b10] Robinson PP (1988). The characteristics and regional distribution of afferent fibres in the chorda tympani of the cat. J Physiol.

[pgen-0010003-b11] Boudreau JC, White TD, Bullard RW (1978). Flavor chemistry of carnivore taste system.

[pgen-0010003-b12] Beidler LM, Fishman IY, Hardiman CW (1955). Species differences in taste responses. Am J Physiol.

[pgen-0010003-b13] Bachmanov AA, Li X, Reed DR, Ohmen JD, Li S (2001). Positional cloning of the mouse saccharin preference (Sac) locus. Chem Senses.

[pgen-0010003-b14] Max M, Shanker YG, Huang L, Rong M, Liu Z (2001). Tas1r3, encoding a new candidate taste receptor, is allelic to the sweet responsiveness locus Sac. Nat Genet.

[pgen-0010003-b15] Nelson G, Chandrashekar J, Hoon MA, Feng L, Zhao G (2002). An amino-acid taste receptor. Nature.

[pgen-0010003-b16] Nelson G, Hoon MA, Chandrashekar J, Zhang Y, Ryba NJ (2001). Mammalian sweet taste receptors. Cell.

[pgen-0010003-b17] Li X, Staszewski L, Xu H, Durick K, Zoller M (2002). Human receptors for sweet and umami taste. Proc Natl Acad Sci U S A.

[pgen-0010003-b18] Montmayeur JP, Matsunami H (2002). Receptors for bitter and sweet taste. Curr Opin Neurobiol.

[pgen-0010003-b19] Liao J, Schultz PG (2003). Three sweet receptor genes are clustered in human chromosome 1. Mamm Genome.

[pgen-0010003-b20] Murphy WJ, Sun S, Chen Z, Yuhki N, Hirschmann D (2000). A radiation hybrid map of the cat genome: Implications for comparative mapping. Genome Res.

[pgen-0010003-b21] Pin JP, Galvez T, Prezeau L (2003). Evolution, structure, and activation mechanism of family 3/C G-protein-coupled receptors. Pharmacol Ther.

[pgen-0010003-b22] Rajavel KS, Neufeld EF (2001). Nonsense-mediated decay of human HEXA mRNA. Mol Cell Biol.

[pgen-0010003-b23] Flynn JJ, Nedbal MA (1998). Phylogeny of the Carnivora (Mammalia): congruence vs incompatibility among multiple data sets. Mol Phylogenet Evol.

[pgen-0010003-b24] Mattern MY, McLennan DA (2000). Phylogeny and speciation of Felids. Cladistics.

[pgen-0010003-b25] Grace J, Russek M (1968). The influence of previous experience on the taste behavior of dogs toward sucrose and saccharin. Physiol Behav.

[pgen-0010003-b26] Kim U, Jorgenson E Coon H, Leppert M, Risch N (2003). Positional cloning of the human quantitative trait locus underlying taste sensitivity to phenylthiocarbamide. Science.

[pgen-0010003-b27] Dahanukar A, Foster K, van der Goes van Naters WM, Carlson JR (2001). A Gr receptor is required for response to the sugar trehalose in taste neurons of Drosophila. Nat Neurosci.

[pgen-0010003-b28] Li X, Inoue M, Reed DR, Huque T, Puchalski RB (2001). High-resolution genetic mapping of the saccharin preference locus (Sac) and the putative sweet taste receptor (T1R1) gene (Gpr70) to mouse distal Chromosome 4. Mamm Genome.

[pgen-0010003-b29] Li X, Bachmanov AA, Li S, Chen Z, Tordoff MG (2002). Genetic, physical, and comparative map of the subtelomeric region of mouse Chromosome 4. Mamm Genome.

[pgen-0010003-b30] Kitagawa M, Kusakabe Y, Miura H, Ninomiya Y, Hino A (2001). Molecular genetic identification of a candidate receptor gene for sweet taste. Biochem Biophys Res Commun.

[pgen-0010003-b31] Montmayeur JP, Liberles SD, Matsunami H, Buck LB (2001). A candidate taste receptor gene near a sweet taste locus. Nat Neurosci.

[pgen-0010003-b32] Sainz E, Korley JN, Battey JF, Sullivan SL (2001). Identification of a novel member of the T1R family of putative taste receptors. J Neurochem.

[pgen-0010003-b33] Reed DR, Li S, Li X, Huang L, Tordoff MG (2004). Polymorphisms in the taste receptor gene (Tas1r3) region are associated with saccharin preference in 30 mouse strains. J Neurosci.

[pgen-0010003-b34] Lu K, McDaniel A, Tordoff M, Li X, Beauchamp G (2005). No relationship between sequence variation in protein coding regions of the Tas1r3 gene and saccharin preference in rats. Chem Senses.

[pgen-0010003-b35] Gilad Y, Man O, Paabo S, Lancet D (2003). Human specific loss of olfactory receptor genes. Proc Natl Acad Sci U S A.

[pgen-0010003-b36] Young JM, Friedman C, Williams EM, Ross JA, Tonnes-Priddy L (2002). Different evolutionary processes shaped the mouse and human olfactory receptor gene families. Hum Mol Genet.

[pgen-0010003-b37] Gilad Y, Wiebe V, Przeworski M, Lancet D, Paabo S (2004). Loss of olfactory receptor genes coincides with the acquisition of full trichromatic vision in primates. PLoS Biol.

[pgen-0010003-b38] Parry CM, Erkner A, le Coutre J (2004). Divergence of T2R chemosensory receptor families in humans, bonobos, and chimpanzees. Proc Natl Acad Sci U S A.

[pgen-0010003-b39] Schaeren-Wiemers N, Gerfin-Moser A (1993). A single protocol to detect transcripts of various types and expression levels in neural tissue and cultured cells: In situ hybridization using digoxigenin-labelled cRNA probes.

[pgen-0010003-b40] Grosvenor W, Kaulin Y, Spielman AI, Bayley DL, Kalinoski DL (2004). Biochemical enrichment and biophysical characterization of a taste receptor for L-arginine from the catfish, Ictalurus puntatus. BMC Neurosci.

